# A case of renal artery thrombosis with renal infarction

**DOI:** 10.4103/0974-2700.66569

**Published:** 2010

**Authors:** Valerie M Lopez, Jonathan Glauser

**Affiliations:** Emergency Services Institute, Cleveland Clinic, Cleveland, OH, USA

**Keywords:** Flank pain, renal infarction, thrombosis

## Abstract

Renal artery thrombosis is a rare, but serious and often misdiagnosed, condition. Emergency physicians and other physicians need to consider this diagnosis in unexplained flank pain, especially in patients with risk factors for this disease. In this case report, the authors review a case of renal infarction caused by renal artery thrombosis in a patient with risk factors for thrombosis but no previous history of thromboembolism. A review of scholarly articles was performed and the case is discussed in the context of the current knowledge of this condition. Common presenting symptoms, features of the history and risk factors will all be discussed herein. Diagnostic evaluation of flank pain in the setting of the suspicion of renal infarction will be discussed, including the modalities of high-resolution computed tomography, renal angiography, scintography and ultrasound. Acute management and prognosis will also be discussed.

## INTRODUCTION

Renal artery thrombosis is a rare, but serious and often misdiagnosed, condition. In a series of over 14,000 autopsies, about 200 cases of kidney infarction were identified – a prevalence of 14/1000.[1] The largest case series of emergency department patients give a prevalence of 0.02/1000,[2,3] with <50 patients in each series.

This diagnosis should be considered in the setting of undiagnosed flank pain, especially in patients with risk factors for this disease. We will present a case of flank pain presenting for emergency evaluation in an emergency department and then discuss the evaluation and treatment options.

## CASE REPORT

A 65-year-old male presented with an abrupt onset of sharp, burning, constant right lower abdominal pain after previously been feeling well. There was no history of trauma, fever, vomiting, diarrhea, chest pain, shortness of breath, syncope, dysuria, hematuria or melena. His past medical history included hypertension, atrial flutter and cardiomyopathy, and he had an implantable cardioverter/defibrillator (ICD). There was no prior history of abdominal aortic aneurysm in the patient or in his family. His past surgical history included an inguinal hernia repair. His medications included aspirin, valsartan, metoprolol and atorvastatin. He did not smoke, use alcohol or illicit drugs.

Physical exam showed vitals signs as follows: body temperature of 36.3°C, blood pressure of 140/82 mmHg, heart rate of 52 beats per minute, respiratory rate of 18 breaths per minute and pulsoximetry of 98% on room air. He was alert, but appeared in mild distress due to pain. His abdominal exam revealed tenderness to palpation in the right lower quadrant, with no rebound, guarding, distention or organomegaly. The remainder of the exam was normal.

The initial emergency department evaluation included an electrocardiogram that showed atrial flutter with a 4:1 block. Laboratory assessment revealed trace hematuria, no leukocytosis, creatinine of 1.3 mg/dL (normal range, 0.7–1.4 mg/dL), mildly elevated lipase to 85 U/L (normal range, 12–70 U/L), mild hyperglycemia and mildly elevated aspartate aminotransferase to 46 U/L (normal range, 7–40 U/L). The lactate and coagulation profiles were within normal limits. Lactate dehydrogenase was not ordered at presentation, but obtained 2 days after admission, and was elevated at 1,479 U/L (normal range, 100–220 U/L). Lactate dehydrogenase is present in the renal tissue and elevates in serum after kidney insult, and it is unknown whether this level correlates to the severity of the infarct.

**Figure 1:**
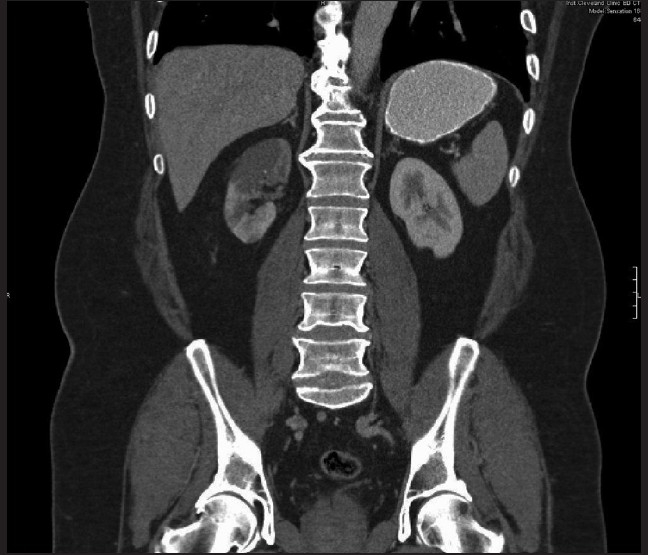
Axial computed tomography enhanced with IV contrast exhibiting minimal enhancement of the right kidney with some spared areas of the periphery, consistent with a cortical rim sign (spared peripheral circulation via the capsular collateral arterioles)

**Figure 2:**
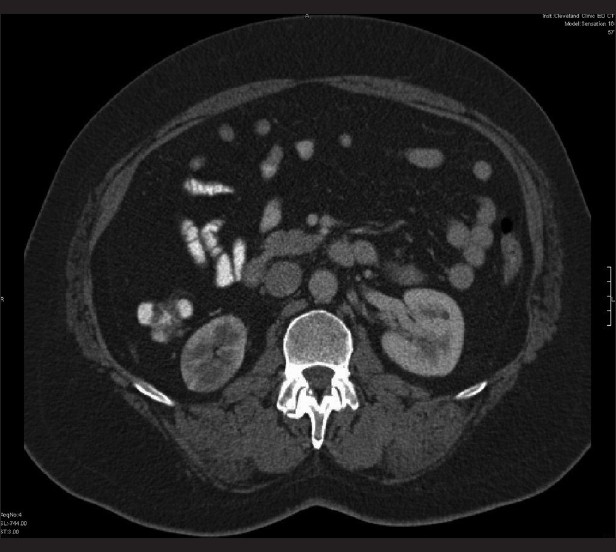
Coronal recon computed tomography enhanced with IV contrast exhibiting an irregular hypodense area of superior pole of the right kidney consistent with hypoperfusion

Given the nature of the pain and the hematuria seen on urinalysis and the history of cardiomyopathy with atrial flutter, both nephrolithiasis and mesenteric ischemia were strongly considered as causes for this acute pain. Therefore, both non-contrast- and contrast-enhanced helical computed tomography (CT) images [Figures 1 and 2] were obtained, which showed extensive infarction involving the upper pole of the right kidney with multiple patchy infarcts into the lower pole, a patent right renal artery, but no visualization of the interparenchymal arterial branches within the mid and upper poles of the right kidney, suggesting arterial thromboembolic disease. There was poor opacification of the right renal vein, and, therefore, renal vein thrombus could not be ruled out. A 2-mm left renal calculus was also identified.

The urology and vascular surgery services were consulted, and both recommended conservative treatment with anticoagulation. In addition to normal saline and dilaudid for pain control, a heparin bolus of 80 mg/kg was given and an infusion at 18 mg/kg/h was initiated, with the patient being admitted to the general medicine service.

It was concluded his renal artery thrombosis was most likely embolic and a 2-D echocardiogram was ordered, which showed no intramural thrombus. He was discharged 5 days later, on coumadin. His creatinine, which was 1.3 mg/dL on admission, rose to 1.8 mg/dL and fell to 1.7 mg/dL by discharge. One month later, the creatinine levels had declined to 1.6 mg/dL.

## DISCUSSION

### Pathogenesis/Risk Factors

Renal infarct is commonly believed to be an under-diagnosed cause of acute abdominal/flank pain.[1–3] It is typically caused by blood or cholesterol clots occluding the renal artery or branch vessels. The source of blood clots, typically, is from atrial fibrillation causing thrombogenesis in the left atrium and left atrial appendage. Cholesterol-based emboli cause partial occlusions with a more chronic presentation. In a study of over 600 patients with peripheral arterial thromboembolism, the most common site of peripheral arterial thromboembolism was the extremities (61%), followed by the mesenteric arteries (29%), the pelvic arteries (9%), the aorta (7%) and, followed last, by the renal arteries (2%).[4] In a case series of 27 patients, 41% had obvious cardiac disease, almost all with atrial fibrillation; however, 59% had no discernible structural or arrhythmic cardiac disease.[5] Idiopathic patients are typically younger[5] and have a paucity of thrombogenic risk factors.[6]

Complete infarct (involving entire kidney due to occlusion or interruption of the renal artery) is the most rare, seen typically after trauma or interventions involving the aorta.[7] Most kidney infarction results from emboli caused by atrial fibrillation or endocarditis,[8] causing only partial occlusion of the renal artery or a branch. Rare causes reported include spontaneous renal artery dissection,[9] dilated cardiomyopathy,[10] paradoxical embolism[11,12] and involved multiple organs. Bilateral infarction has also been reported,[3] including bilateral (global) renal infarction subsequent to dissecting aneurysms of the aorta,[13] with septic emboli from endocarditis, lupus vasculitis or with sickle cell disease[14] or fibromuscular dysplasia of the renal arteries.[15,16] Reports of other causes associated with renal infarction include trauma, vasculitis, instrumentation, transplant, sepsis,[14] sickle cell disease[14,17] and antiphospholipid antibody syndrome,[18,19] cocaine use,[20] and subsequent to carotid artery dissection.[21]

### Clinical presentation of renal infarct

The most common complaints at presentation are flank pain[3,5] or abdominal pain,[22] usually constant in nature.[5] Other common features include fever,[3,5,22] nausea and vomiting.[3,5,22] Lumbar and flank tenderness[5] are seen and are even more likely in the idiopathic/younger group. Oliguria is rarely found.[22] New-onset hypertension is uncommon, but can be seen especially if there are underlying renal artery lesions.[8]

The typical patient tends to be older, with an average age of 67 in one series,[3] although significant portions are younger. Reviewing 89 cases from the literature,[2,3,22,23] the mean age at presentation was 65.7 years, with no significant gender predominance or right or left kidney predominance. About 10% of the cases presented with bilateral renal involvement. Previous thromboembolic events in 20/89 (22%) were observed.

### Differential diagnosis

The differential diagnosis for this disorder, as with all abdominal pain disorders, is extensive. Emergent conditions that will need to be ruled out include causes of acute surgical abdomen, notably appendicitis, diverticulitis, ruptured abdominal aortic aneurysm, testicular/ovarian torsion, incarcerated hernia, intestinal obstruction and/or perforation. Other non-surgical causes such as mesenteric ischemia, nephrolithiasis and pyelonephritis should also be considered. Because of the vague clinical presentation, diagnosis is not often made on admission (only 40% of the patients in one case series)[22] and is delayed often up to 2 days or more.[7] However, it is likely that this accuracy will increase with the increasing availability of computed tomographic (CT) scans, which are the best way to recognize an infarct.

### Evaluation

#### Labs

Urinalysis is recommended, with hematuria being a very common finding, seen in all cases in one study.[3] However, hematuria is not universal, with about one-half of the patients in another study showing no hematuria,[22] and two cases from another center also with no hematuria.[24] In contrast, elevations of lactate dehydrogenase (LDH) are almost universal, with nearly all patients in one study[5] and in all cases in other reports.[3,24] Proteinuria was seen in 45% of the patients in one retrospective case review.[22] The white blood cell count was often elevated.[3,5] Serum creatinine was not markedly elevated at presentation, but a small increase was seen[5] at peak in one study and a more marked elevation was seen in another slightly larger case review.[22] C-reactive protein is also often elevated.[5]

#### Diagnostic imaging

Angiography is positive in 100% of the cases (10/10).[22] Renal isotope exam is abnormal in 97%.[22] CT scan is diagnostic in 80% of the cases.[22] Ultrasound is positive in only 3% of the cases.[22] Contrast CT is the diagnostic modality of choice at this time, with the cardinal findings of a wedge-shaped, peripheral, non-enhancing area. The most common finding is a hypoattenuated area with associated mass effect,[22] sometimes accompanied by a cortical rim sign. The cortical rim sign represents opacification of a rim of functioning nephrons supplied via capsular collaterals surrounding an otherwise non-functioning kidney.[25] The cortical rim sign can be especially useful in differentiating ischemia (where it is sometimes seen, especially with global infarcts) vs. pyelonephritis (where it is not seen).[14] Wedge-shaped focal infarcts, global infarcts and multifocal infarcts can also be seen with infarction.[26] In a series of 12 patients, 47% had a cortical rim sign, 21% had a subcapsular fluid collection, 11% had a mass effect and 6% had abnormally thickened renal fascia.[14]

Because of the vague presentation and the lack of easily obtainable definitive imaging, high clinical suspicion will need to be maintained in order to achieve diagnosis. The improvement of the CT technique and increasing utilization will likely improve upon the historical diagnostic accuracy. However, the use of non-contrast-enhanced scan is increasingly thought to be capable of diagnosing not only renal calculi but also appendicitis, diverticulitis, biliary tract disease, some aortic conditions and gynecologic diseases, but is notably unable to detect thromboembolic and other renal vascular disease.[25]

Because non-contrast CT is often the study of choice, when is a contrast-enhanced study appropriate? Some general recommendations proposed include the presence of unilateral perinephric stranding without hydrouteronephrosis (suspicious for renal infarction, renal vein thrombosis and pyelonephritis), the presence of significant hypo/hyperdense perirenal collections (urinoma, hematoma), the presence of a mass or complicated cyst and negative unenhanced CT in a patient with unexplained hematuria.[27]

Additionally, even if a contrast-enhanced study is ordered, the sensitivity is only 80%. Therefore, if the patient is at risk for embolic events and has an elevated serum LDH, it is recommended to further image with renal isotope exam (scintography) or with renal angiography[22] if the contrast-enhanced CT is negative.

### Treatment

Because of the infrequent nature of this malady, no large prospective studies have evaluated the optimal treatment modalities regarding the dosing of heparin, the use of low molecular weight heparin or thrombolytics or using medical management vs. surgical management. At this time, there are only recommendations based on the consensus in the literature.

1. Anticoagulation: Start with a heparin bolus with an infusion followed by initiation of coumadin therapy indefinitely, which has been recommended.[3,22] The dosing of initial heparin as well as the target International Normalized Ratio (INR) and the length of coumadin therapy has not been defined. Anticoagulation targets with the usual 2.0–3.0 INR are recommended unless the patient thrombosed while therapeutic, in which case a target of 2.5–3.5 may be more desirable. Many patients will remain on coumadin indefinitely as they have an underlying pro-embolic condition such as atrial fibrillation.
2. Antihypertensive: Patients with prior hypertension or with new-onset hypertension from the infarct should be treated with an anti-hypertensive.[8] Because of the insult to the kidney, hypertension often develops and because this is mediated through the increase of renin, it is thought that using angiotensin-converting enzyme inhibitors or an angiotensin receptor blocker may be the most suitable.
3. Thrombolysis/thrombectomy: These interventions are currently supported only by case reports, which report successful reperfusion but not uniform improvement in renal outcome. Open surgery is not recommended other than in the case of trauma, where other problems may indicate the need for surgery anyway. In one case report, embolectomy has been performed 30 h after complete bilateral occlusion with complete resolution of kidney failure.[28] Local thrombolysis or thrombectomy with minimally invasive percutaneous endovascular therapy for acute occlusions of the main renal artery or significant branch can be considered but, again, no significant clinical evidence supports this intervention.

### Prognosis

The most common sequel to renal infarction is loss of renal function and persistent hypertension.[8] However, many patients go on to have normal kidney function[3,8,22] with no permanent hypertension. A small percent will need dialysis, 8% in one case series.[22]

Repeat embolic events have been observed, some with events prior to the renal infarct and some with subsequent events.[22] Because of the lack of prospective study data, it is unknown what the benefit of continuing anticoagulation would be. However, given data regarding other catastrophic thrombotic events, it is likely recommended to continue indefinitely in the setting of atrial fibrillation.

## SUMMARY

In general, acute kidney infarction is rare,[8] and perhaps because of this as much as its non-specific presentation, it is often missed on presentation. When evaluating a patient with known atrial fibrillation, emergency physicians typically think of ischemic stroke as the usual arterial thrombotic/embolic sequelae of this syndrome. But, there are other embolic syndromes which, although rare, should also be considered in patients with this particular comorbidity. Renal infarction should be strongly considered when presented with the following triad: persistent abdominal and/or flank pain, elevated serum LDH and/or hematuria and risk of thromboembolic event,[5] with the caveat that recent evidence,[5] likely aided by the increased diagnostic accuracy of CT, seem to reveal a significant portion of patients with this disease missing the thrombotic part of this triad. Consider enhanced CT, especially in patients with elevated LDH, and if this is negative, consider follow-up with radioisotope renal scan and evaluating for atrial thrombus.
